# Toll Like Receptor 2, 4, and 9 Signaling Promotes Autoregulative Tumor Cell Growth and VEGF/PDGF Expression in Human Pancreatic Cancer

**DOI:** 10.3390/ijms17122060

**Published:** 2016-12-08

**Authors:** Tanja Grimmig, Romana Moench, Jennifer Kreckel, Stephanie Haack, Felix Rueckert, Roberta Rehder, Sudipta Tripathi, Carmen Ribas, Anil Chandraker, Christoph T. Germer, Martin Gasser, Ana Maria Waaga-Gasser

**Affiliations:** 1 Department of Surgery I, Molecular Oncology and Immunology, University of Wuerzburg, 97080 Wuerzburg, Germany; Grimmig_T@ukw.de (T.G.); Moench_R@ukw.de (R.M.); Jennifer.Kreckel@uni-wuerzburg.de (J.K.); stephanie-haack@web.de (S.H.); 2 Surgical Clinic Mannheim, University of Heidelberg, 68167 Mannheim, Germany; felix.rueckert@umm.de; 3 Medical School, Evangelic Faculty of Paraná, 80730-000 Curitiba, Brazil; robertarehder1@gmail.com (R.R.); marcondes.ribas@gmail.com (C.R.); 4 Brigham and Women’s Hospital, Transplant Research Center, Harvard Medical School, Boston, MA 02115, USA; STRIPATHI@PARTNERS.ORG (S.T.); ACHANDRAKER@PARTNERS.ORG (A.C.); 5 Department of Surgery I, University of Wuerzburg, 97080 Wuerzburg, Germany; Germer_c@ukw.de (C.T.G.); gasser_m@ukw.de (M.G.)

**Keywords:** TLR2, TLR4, TLR9, pancreatic cancer, inflammation, tumor growth

## Abstract

Toll like receptor (TLR) signaling has been suggested to play an important role in the inflammatory microenvironment of solid tumors and through this inflammation-mediated tumor growth. Here, we studied the role of tumor cells in their process of self-maintaining TLR expression independent of inflammatory cells and cytokine milieu for autoregulative tumor growth signaling in pancreatic cancer. We analyzed the expression of TLR2, -4, and -9 in primary human cancers and their impact on tumor growth via induced activation in several established pancreatic cancers. TLR-stimulated pancreatic cancer cells were specifically investigated for activated signaling pathways of VEGF/PDGF and anti-apoptotic Bcl-xL expression as well as tumor cell growth. The primary pancreatic cancers and cell lines expressed TLR2, -4, and -9. TLR-specific stimulation resulted in activated MAP-kinase signaling, most likely via autoregulative stimulation of demonstrated TLR-induced VEGF and PDGF expression. Moreover, TLR activation prompted the expression of Bcl-xL and has been demonstrated for the first time to induce tumor cell proliferation in pancreatic cancer. These findings strongly suggest that pancreatic cancer cells use specific Toll like receptor signaling to promote tumor cell proliferation and emphasize the particular role of TLR2, -4, and -9 in this autoregulative process of tumor cell activation and proliferation in pancreatic cancer.

## 1. Introduction

Pancreatic ductal adenocarcinoma (PDAC) is the most lethal type of digestive cancer with a five-year survival rate of about 7%. In contrast to increasing survival rates for patients with various other cancer types, therapeutic improvements over the last four decades in pancreatic cancer raised the chances for recovery only about 3% [1]. Besides advanced age, smoking, obesity and diabetes, chronic pancreatitis is a major risk factor for the development of PDAC [2]. During chronic pancreatitis, the pancreas undergoes damaging events promoted through inflammation processes which result in the release of damage-associated molecular patterns (DAMPs) as well as growth factors like vascular endothelial growth factor (VEGF) during subsequent healing [3,4].

In addition to a panel of pathogen-associated molecular patterns (PAMPs) including cell-wall components like lipopolysaccharide (LPS) as well as microbial DNA and RNA, DAMPs which arise from inflammation and cellular injury can stimulate TLRs and consequently induce TLR signaling that supports an inflammatory microenvironment [5,6]. Recently, enhanced expression of TLRs has been described in a variety of different tumor entities and depending on the cancer type, could be linked to either favorable or poor prognosis [7,8,9,10,11]. TLR3 and -4 were identified as predictors of poor survival in breast cancer, whereas high TLR9 predicted enhanced survival [12,13]. In esophageal cancer TLR3, -4, -7, and -9 overexpression has been demonstrated in 70%, 72%, 67%, and 78% of patient tumors, respectively, and, as in breast cancer, poor prognosis was associated with elevated TLR3 expression on tumor cells, however, patients expressing increased TLR9 associated with fibroblasts exhibited improved survival [12,13,14]. Enhanced colorectal tumor expression of TLR7/8 colocalized with the cancer stem cell marker CD133 and correlated with reduced overall survival [7,13]. In one report, enhanced TLR4 expression was identified in 69% of patients with pancreatic cancer and correlated with increased NF-κB signaling, enhanced hypoxia-inducible factor-1α (HIF-1α) expression, and dramatically reduced patient survival [13,15].

In controversy to studies examining the anti-tumor effects of TLR signaling, several tumor entities, particularly those linked to inflammatory processes, have demonstrated unfavorable effects of TLR activation. TLR7 ligands are currently in use for the treatment of various cancer types such as melanoma, breast cancer and basal cell carcinoma [16]. Moreover, TLR3 and -9 activation has been demonstrated to reduce tumor growth in renal cell carcinoma and metastatic colorectal cancer [16,17] Besides those data that demonstrate favorable effects of TLR activation it is known that TLR ligands can also promote cancer cell survival, migration and tumor progression. For example, TLR agonists have been shown to induce tumor cell viability and metastasis in human lung cancer [18]. Activation of TLR3 resulted in increased proliferation in human myeloma cells [19]. Additionally, TLR ligation is related to adhesion and metastasis in human colorectal cancer cells and migration in human glioblastoma (via TLR4) and human breast cancer cells (via TLR2) [18,20,21]. Previously, we showed that TLR7 or -8 expression is associated with UICC stage in PDAC and stimulation increases tumor cell proliferation and resistance to the cytostatic agent 5-fluorouracil (5-FU) in pancreatic cancer cells [8].

Next to TLRs, it is well-known that the expression of growth factors such as VEGF or platelet-derived growth factor (PDGF) is increased in various tumors. Additionally, growth factor expression is associated with augmented angiogenesis and tumor cell proliferation and therefore linked to poor outcome [22,23,24].

In this study, we analyzed the expression of TLR2, -4, and -9 in pancreatic cancer. Besides their pathogen-associated ligands (TLR2: multiple lipoproteins and lipoteichoic acid; TLR4: LPS; and TLR9: unmethylated CpG oligodeoxynucleotide DNA), these TLRs are also known to be induced by several endogenous ligands such as heat shock proteins, fibrinogen, hyaluronic acid fragments and high-mobility group box 1 (HMGB1) [25]. These ligands arise from damaging events promoted through inflammatory processes [3]. Consequently, TLR2, -4, and -9 are highly suspected to play a role in inflammation linked cancers such as pancreatic cancer.

To examine the impact of TLR2, -4, and -9 expression and activation in pancreatic cancer cells, effects of TLR agonists CpG oligonucleotides 2006 (ODN, TLR9 specific), lipoteichnic acid of *Staphylococcus aureus* (LTA, TLR2 specific), lipopolysaccharide (LPS, TLR4 specific), and HMGB1 (non-specific) on growth factor expression, tumor cell signaling and cancer proliferation were analyzed to elucidate the potential of TLR signaling as a target for therapeutic strategies in PDAC.

## 2. Results

### 2.1. TLR2, -4, and -9 Are Expressed in Human Pancreatic Cancer Tissue 

**Figure 1:**
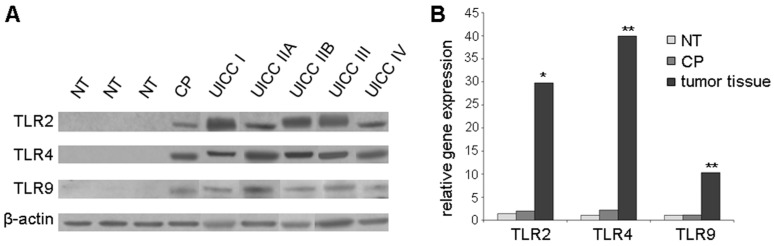
Increased TLR2, -4, and -9 expression in tissues of chronic pancreatitis and pancreatic cancer: (**A**) Representative examples of Western blot analysis of normal pancreatic tissue (NT), tissue from chronic pancreatitis (CP), and primary pancreatic cancer at all stages (UICC I, IIA, IIB, III, IV). β-actin probe was used as a control for protein loading; (**B**) RT-qPCR of normal pancreatic tissue (NT, *n* = 4), tissue from chronic pancreatitis (CP, *n* = 4), and primary pancreatic tumor tissue at UICC stages II and III (*n* = 14). Values for normal pancreatic tissue were standardized to baseline. The relative gene expression is expressed as 2^−ΔΔ*C*q^ (fold difference, FD). * *p* < 0.05, ** *p* < 0.005.

Western blot analysis of pancreatic tissue showed no protein expression of TLR2, -4, and -9 in normal pancreatic tissue (NT) compared to increased expression in tissue of chronic pancreatitis (CP) and in particular in primary pancreatic cancer at all stages (UICC I, IIA, IIB, III, and IV) (Figure 1A).

In RT-qPCR, elevated relative gene expression of TLR2 (fold difference, FD = 29.8, *p* < 0.05), TLR4 (FD = 39.9, *p* < 0.005), and TLR9 (FD = 10.3, *p* < 0.005) was observed in pancreatic tumor tissues compared to normal tissues (Figure 1B). Additionally, TLR2 and -4 gene expression was not significantly increased in tissue of chronic pancreatitis compared to normal tissue (TLR2 FD = 2.0 and TLR4 FD = 2.2, respectively) (Figure 1B).

**Figure 2:**
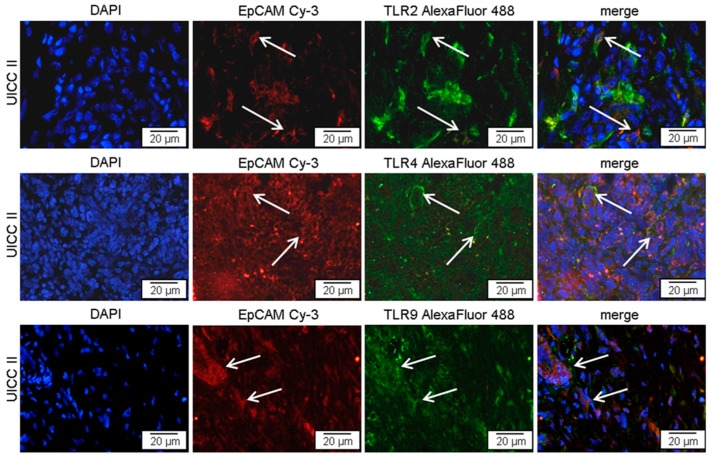
TLR2, -4, or -9 expressing tumor cells in pancreatic cancer tissue. Representative examples of immunofluorescence double staining, showing TLR (green) and EpCAM (red) co-staining (arrows) in tumor cells of patients with pancreatic cancer UICC II. AlexaFluor 488, green; Cy3 (indocarbocyanin), red; DAPI (49,6-diamidino-2-phenylindoldihydrochlorid), blue—nuclear counterstaining.

To substantiate that elevated TLR expression found in ex vivo pancreatic cancer tissue by Western blot and RT-qPCR is associated with pancreatic cancer cells rather than tumor infiltrating cells of the immune system, immunofluorescence double staining of cryo sections was performed. Co-staining of TLR2, -4, and -9 with the epithelial marker EpCAM clearly indicated TLR expressing tumor cells in primary tumor tissue of all UICC stages (data not shown). In Figure 2 representative specimens for TLR2, -4, and -9 staining in pancreatic tumor tissues at UICC stage II are demonstrated and examples for TLR and EpCAM co-expressing cells are marked with white arrows.

### 2.2. TLR2, -4, and -9 Are Expressed in Human Pancreatic Cancer Cell Lines

Expression of TLR2, -4, and -9 was analyzed by RT-qPCR and Western blot in five established human pancreatic cancer cell lines (Panc1, MIAPaCa-2, BxPC-3, AsPC-1, and SW1990) as well as in three primary human pancreatic cancer cell lines (PaCaDD135, PaCaDD159, and PaCaDD185). TLR mRNA was detected in all investigated cell lines indicating constitutive expression of TLR2, -4, and -9 in unstimulated human pancreatic cancer cells. To allow for comparison of RT-qPCR results, cell lines with the lowest expression were standardized to baseline (fold difference, FD = 1).

**Figure 3:**
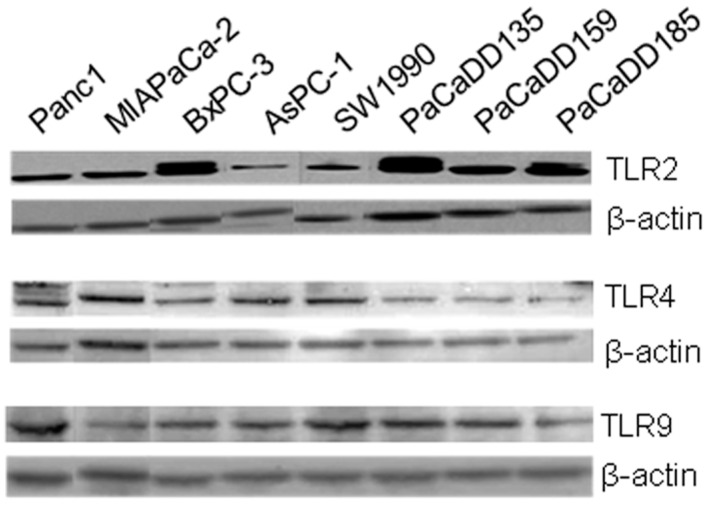
Western blot analysis of TLR2, -4, and -9 expression in human pancreatic cancer cell lines. TLR2, -4, and -9 protein expression was detected in all investigated established pancreatic cancer cell lines (Panc1, MIAPaCa-2, BxPC-3, AsPC-1, and SW1990) and primary pancreatic cancer cell lines (PaCaDD135, PaCaDD139, and PaCaDD185). β-actin probe was used as a control for protein loading.

For TLR2, expression range was observed from FD = 1 (AsPC-1 and MIAPaCa-2) to FD < 40 (PaCaDD135) (Figure 3A). Besides Panc1 (FD = 11), five out of eight cell lines demonstrated expression levels FD > 40 (PaCaDD185, PaCaDD159, SW1990, BxPC-3, and PaCaDD135) (data not shown). As observed for TLR2, MIAPaCa-2 cells also demonstrated lowest TLR4 gene expression and FD value was therefore standardized to baseline (FD = 1). mRNA levels in further analyzed cell lines varied from four-fold (SW1990 FD = 4) to >40-fold (PaCaDD159). Again, five out of eight cell lines showed considerably pronounced expression (FD > 40): BxPC-3, PaCaDD185, PaCaDD135, AsPC-1, and PaCaDD159 (data not shown). For TLR9, expression in BxPC-3 cells were standardized to baseline (FD = 1). Additionally, PaCaDD159 and PaCaDD185 demonstrated identical expression intensity (FD = 1). In the remaining cell lines, expression range was observed from FD = 2 (Panc1) to FD = 26 (PaCaDD135). MIAPaCa-2 cells showed four-fold (FD = 4), AsPC-1 cells six-fold (FD = 6), and SW1990 cells nine-fold (FD = 9) TLR9 expression compared to BxPC-3 cells (data not shown). In general, the range of TLR9 gene expression intensity (FD = 1 to 26) was less distinct than TLR2 (FD = 1 to >40) and TLR4 (FD = 1 to >40).

In Western blot analysis, TLR protein expression was also demonstrated in all investigated pancreatic cancer cell lines. Consistent with gene expression results, most intense TLR2 protein expression was detected in BxPC-3 and PaCaDD135 cells (Figure 3). TLR4 exhibited highest protein amounts in MIAPaCa-2 and SW1990 cells (next to AsPC-1 and Panc1) while BxPC-3 and primary pancreatic cancer cell lines PaCaDD135, PaCaDD159, and PaCaDD185 demonstrated only moderate TLR4 expression (Figure 3). For TLR9, most intense protein expression was observed in cell lines Panc1, SW1990, and PaCaDD135 (Figure 3).

Based on cell line characteristics and TLR expression profile, three out of eight cell lines were chosen for further in vitro analysis: MIAPaCa-2 (established cell line, originated from primary tumor, showing moderate TLR expression), BxPC-3 (established cell line, originated from primary tumor, showing intense TLR expression), and PaCaDD135 (primary cell line, showing intense TLR expression).

**Figure 4:**
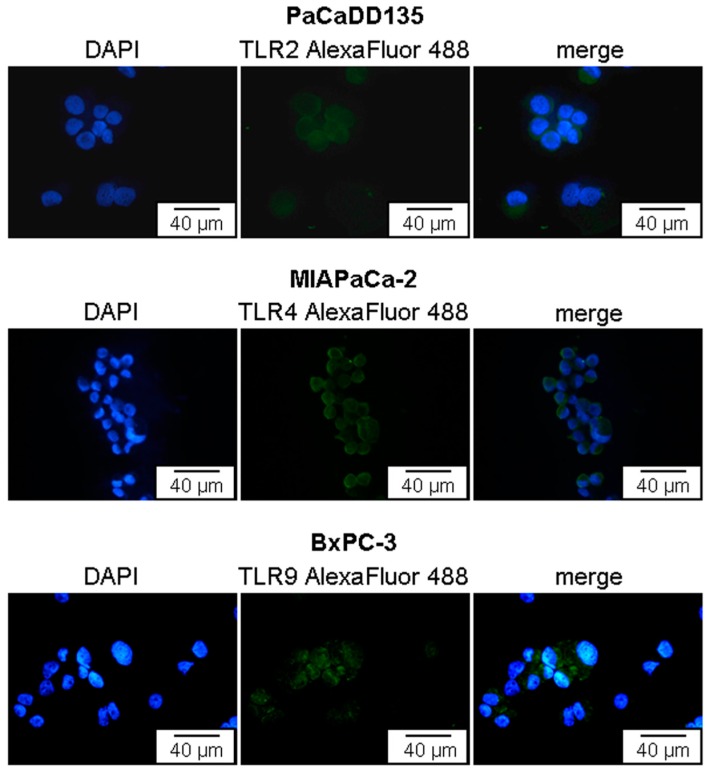
Immunofluorescent staining of TLR2, -4, and -9 in PaCaDD135, MIAPaCa-2, and BxPC-3 pancreatic cancer cells. Representative examples showing TLR2, -4, and -9 expression (green). Cell surface localization of TLR2 and -4 as well as intracellular localization of TLR9 can be identified. AlexaFluor 488, green; DAPI (49,6-diamidino-2-phenylindoldihydrochlorid), blue—nuclear counterstaining.

**Figure 5:**
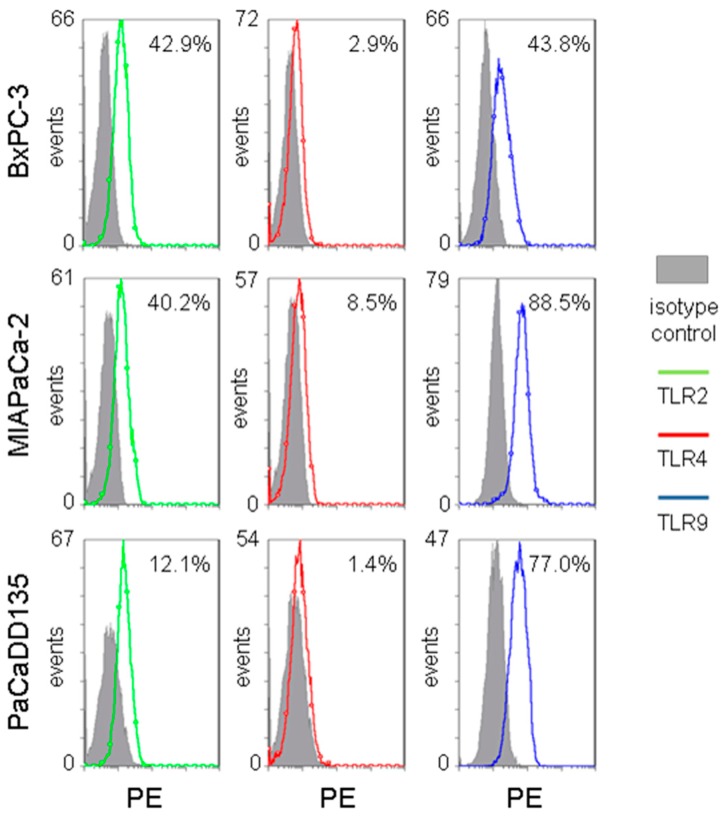
Distribution of TLR2, -4, and -9 expressing cells in pancreatic cancer cell lines. Flow cytometric analysis of BxPC-3, MIAPaCa-2, and PaCaDD135 using PE-conjugated antibodies against TLR2, -4, and -9. TLR2 depicted as green line, TLR4 as red line, and TLR9 as blue line, isotype control as grey area, PE (phycoerythrin).

To investigate the distribution and localization of TLR2, -4, and -9 in selected cell lines, immunostainings were performed. TLR2, -4, and -9 was detected in cytospin preparations of all three examined cancer cell lines (PaCaDD135, MIAPaCa-2 and BxPC-3 cells) (Figure 4). Cell surface localization of TLR2 and -4 as well as intracellular localization of TLR9 can be identified. The proportion of TLR positive cells in each cell line was then determined by FACS analysis. In BxPC-3 and MIAPaCa-2 cancer cells TLR2 and -9 expression clearly dominated (BxPC-3: TLR2 42.9%, TLR4 2.9%, and TLR9 43.8%; MIAPaCa-2 TLR2: 40.2%, TLR4 8.5%, and TLR9 88.5%) (Figure 5, top and center). PaCaDD135 tumor cells expressed predominantly TLR9 and to a lesser extent TLR2 and -4 (77.0%, 12.1% and 1.4% respectively) (Figure 5, bottom).

### 2.3. TLR Activation Results in Increased Growth Factor Expression and Release

**Figure 6:**
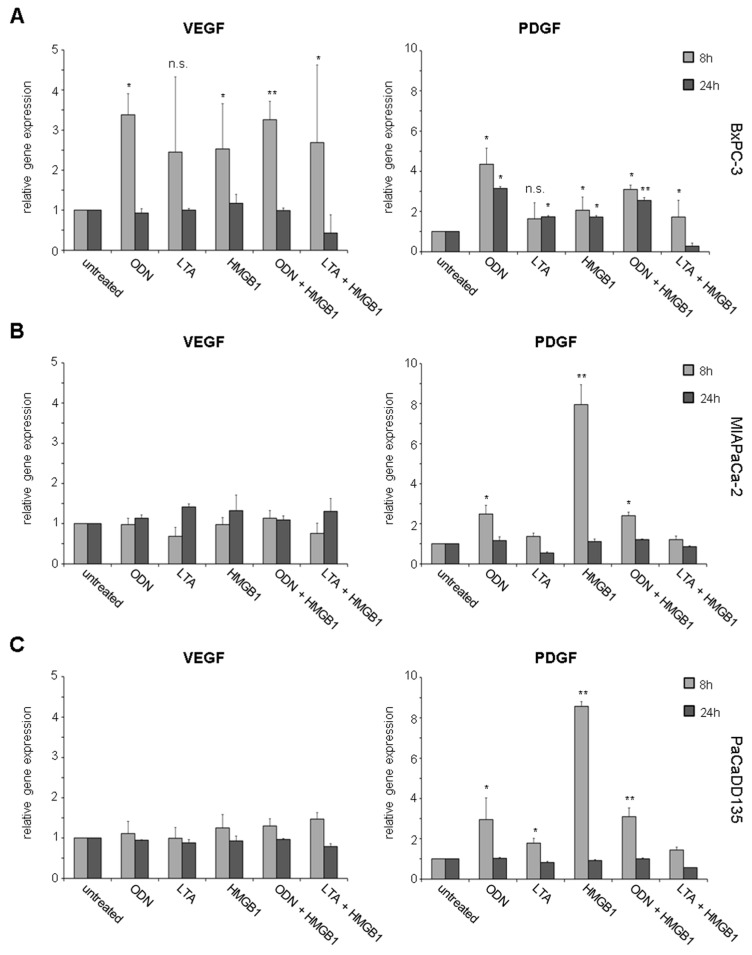
Gene expression analysis of VEGF and PDGF in pancreatic cancer cell lines after treatment with TLR ligands: BxPC-3 (**A**); MIAPaCa-2 (**B**); and PaCaDD135 (**C**) were incubated with ODN, LTA, HMGB1, ODN + HMGB1, and LTA + HMGB1 and analyzed by RT-qPCR 8 and 24 h after stimulation. Increased VEGF gene expression was observed only in BxPC-3 cells, whereas PDGF was significantly expressed in all three cancer cell lines. Untreated cells were standardized to baseline. The relative gene expression is expressed as 2^−ΔΔ*C*q^. * *p* < 0.05, ** *p* < 0.005, n.s. not significant.

Significantly increased VEGF gene expression was observed in BxPC-3 cancer cells when treated with the three TLR ligands either alone or in a combined setting (ODN: TLR9 FD = 3.4, *p* < 0.05; LTA: TLR2 FD = 2.4, n.s.; HMGB1: non-specific FD = 2.5, *p* < 0.05; ODN + HMGB1: FD = 3.3, *p* < 0.005; LTA + HMGB1: FD = 2.7, *p* < 0.05) (Figure 6A, left). After 24 hours of initial TLR ligation relative gene expression dropped to the level of untreated control cells. As compared to VEGF, significantly elevated PDGF gene expression was detected likewise (Figure 6A, right). Eight hours after incubation with the three TLR ligands either alone or combined BxPC-3 cells demonstrated 1.6- to 4.4-fold increase compared to untreated cells (ODN: FD = 4.4, *p* < 0.05; LTA: FD = 1.6, n.s.; HMGB1: FD = 2.1, *p* < 0.05; ODN + HMGB1: FD = 3.1, *p* < 0.05; LTA + HMGB1: FD = 1.7, *p* < 0.05). Interestingly, 24 h after initial treatment with the TLR ligands PDGF expression remained significantly increased (ODN: FD = 3.2, *p* < 0.05; LTA: FD = 1.7, *p* < 0.05; HMGB1: FD = 1.7, *p* < 0.05; ODN + HMGB1: FD = 2.5, *p* < 0.005) (Figure 6A, right).

While in MIAPaCa-2 tumor cells no changes in VEGF gene expression were observed, significantly increased gene expression of PDGF was demonstrated after single or combined stimulation with the TLR ligands (ODN: FD = 2.5, *p* < 0.05; HMGB1: FD = 8.0, *p* < 0.005; ODN + HMGB1: FD = 2.4, *p* < 0.05) (Figure 6B). After 24 h of initial TLR activation relative PDGF gene expression dropped to the level of untreated control cells (Figure 6B, right).

Similar to MIAPaCa-2 cells, no significant effects of TLR activation on VEGF gene expression were detected in PaCaDD135 cancer cells (Figure 6C). In contrast to VEGF, significantly increased PDGF gene expression was demonstrated 8 h after incubation with the three TLR ligands alone or in a combined setting (ODN: FD = 43.0, *p* < 0.05; LTA: FD = 1.7, *p* < 0.05; HMGB1: FD = 8.6, *p* < 0.005; ODN + HMGB1: FD = 3.1, *p* < 0.05). As compared to MIAPaCa-2 cells, PDGF gene expression dropped to the level of untreated control cells 24 h after TLR ligation (Figure 6C, right).

**Figure 7:**
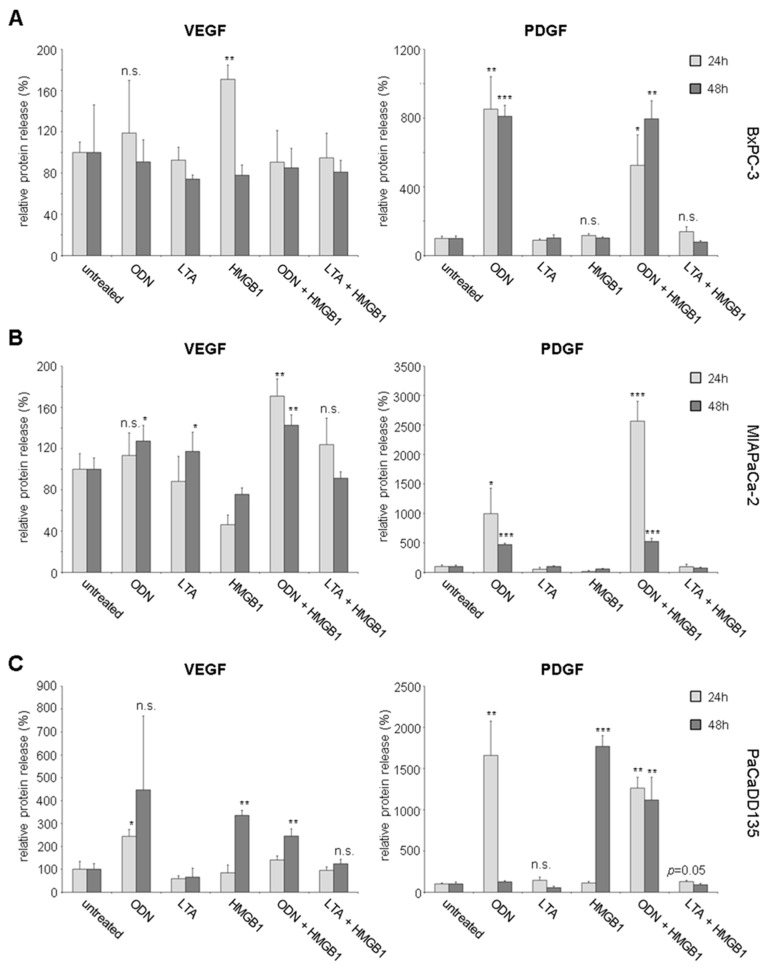
Luminex analysis of VEGF and PDGF expression in supernatants of human pancreatic cancer cells after treatment with TLR ligands: BxPC-3 (**A**); MIAPaCa-2 (**B**); and PaCaDD135 (**C**) were incubated with ODN, LTA, HMGB1, ODN + HMGB1, and LTA + HMGB1 and supernatants were analyzed 24 h and 48 h after stimulation. Untreated cells were standardized to baseline. * *p* < 0.05, ** *p* < 0.005, *** *p* < 0.0001, n.s. not significant.

Next to gene expression analysis, TLR induced release of VEGF and PDGF was examined by Luminex analysis in cell culture supernatants after treatment with TLR agonists. Increased VEGF levels were detected in BxPC-3 cell supernatants 24 h after treatment with two of the TLR ligands (ODN: 119%, and HMGB1: 171%, *p* < 0.005) (Figure 7A, left). PDGF levels were likewise increased after treatment with two of the three TLR ligands either alone or in a combined setting (ODN: 852%, *p* < 0.005; HMGB1: 117%; ODN + HMGB1: 525%, *p* < 0.05; LTA + HMGB1: 140%) (Figure 7A, right). Interestingly, PDGF levels remained highly upregulated 48 h after treatment with the TLR ligand ODN either alone or in a combined setting (ODN: 810%, *p* < 0.0001 and ODN + HMGB1: 800%, *p* < 0.005). MIAPaCa-2 cells demonstrated increased VEGF release 24 h after TLR ligation with ODN (113%), ODN + HMGB1 (171%, *p* < 0.005), and LTA + HMGB1 (124%) (Figure 7B, left). This was continued significantly after 48 h. MIAPaCa-2 cancer cells showed increased VEGF levels after initial TLR ligation either with ODN or LTA alone or combined with ODN and HMGB1 (ODN: 127%, *p* < 0.05; LTA: 117%, *p* < 0.05, and ODN + HMGB1: 143%, *p* < 0.005). For PDGF highly increased levels in supernatants were comparably demonstrated 24 h after stimulation with ODN (994%, *p* < 0.05) and particularly when incubated with both TLR ligands ODN + HMGB1 (2565%, *p* < 0.0001) (Figure 7B, right). After 48 h further prolonged and highly significant PDGF levels were observed (ODN: 470%, *p* < 0.0001 and ODN + HMGB1: 522%, *p* < 0.0001 respectively). Increased VEGF levels were also observed in PaCaDD135 cancer cells 24 h after treatment with the TLR ligands (ODN: 244%, *p* < 0.05 and ODN + HMGB1: 140%) (Figure 7C, left).

After 48 h of initial TLR ligation, VEGF levels continued to be enhanced in all tested supernatants (ODN: 447%, HMGB1: 336%, *p* < 0.0005, ODN + HMGB1: 245%, *p* < 0.005, and LTA + HMGB1: 124%). Elevated PDGF levels were likewise detected 24 h after incubation with either one of the TLR ligands alone (ODN: 1661%, *p* < 0.005, LTA: 144%), or in a combined setting of stimulation (ODN + HMGB1: 1262%, *p* < 0.005, and LTA + HMGB1: 128%, *p* = 0.05) (Figure 7C, right). After 48 h of initial stimulation PaCaDD135 cancer cells continued to respond with high PDGF levels (HMGB1: 1768%, *p* < 0.0001 and ODN + HMGB1: 1119%, *p* < 0.005).

### 2.4. TLR Stimulation Leads to the Activation of MAPK Signaling and Increased Expression of Anti-Apoptotic Protein Bcl-xL

**Figure 8:**
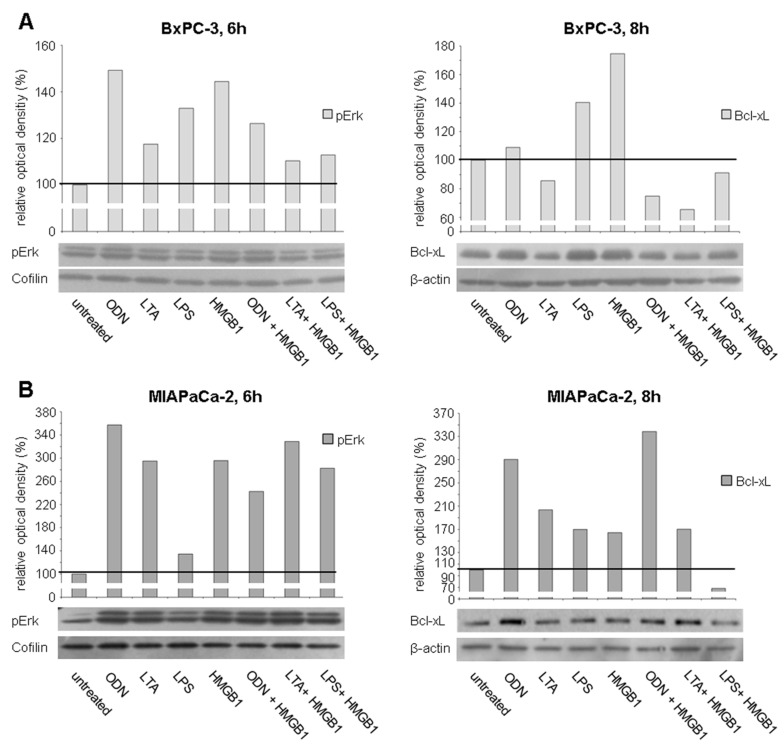

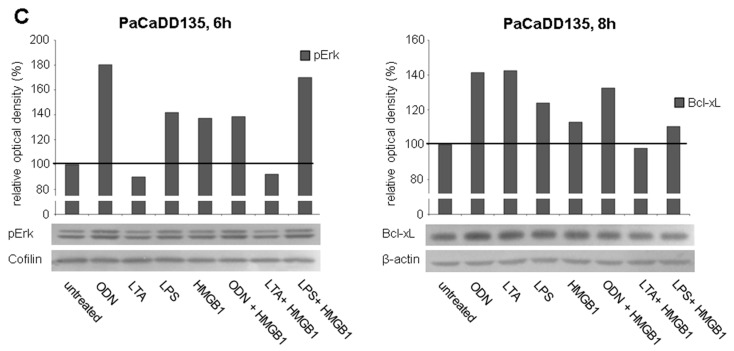
Western blot analysis of pErk and Bcl-xL in different human pancreatic cancer cells after treatment with TLR ligands: BxPC-3 (**A**); MIAPaCa-2 (**B**); and PaCaDD135 cells (**C**) were incubated with ODN, LTA, LPS, HMGB1, ODN + HMGB1, LTA + HMGB1, and LPS + HMGB1 and analyzed 6 h (pErk) and 8 h (Bcl-xL) after stimulation. Cofilin (pErk) and β-actin (Bcl-xL) probes were used as controls for protein loading. Relative optical density (ROD) was determined using ImageJ software. Values for proteins of interest were calculated in relation to values of loading controls. Untreated cells were standardized to baseline.

Phosphorylation of the MAPK pathway component Erk was analyzed after TLR activation by Western blot. In BxPC-3 cancer cells single treatment with TLR agonists either alone or in combination resulted in increased phosphorylation of Erk (ODN: relative optical density, ROD = 149%, LTA: 118%, LPS: 133%, HMGB1: 144%, ODN + HMGB1: 126%, LTA + HMGB1: 110%, and LPS + HMGB1: 113%) (Figure 8A, left). MIAPaCa-2 cells treated with the TLR ligands demonstrated comparably more pronounced pErk activation then BxPC-3 cancer cells (ODN: ROD = 357%, LTA: 295%, LPS: 135%, HMGB1: 296%, ODN + HMGB1: 242%, LTA + HMGB1: 329%, and LPS + HMGB1: 282%). (Figure 8B, left). In PaCaDD135 tumor cells, increased phosphorylation of Erk was observed after treatment with almost all TLR ligands (ODN: 180%, LPS: 142%, HMGB1: 137%, ODN + HMGB1: 139%, and LPS + HMGB1: 170%) (Figure 8C, left). Next to pErk, expression of the anti-apoptotic Bcl-xL protein was examined. In BxPC-3 tumor cells treatment with two of the used TLR ligands as well as LPS resulted in increased Bcl-xL expression (ODN: ROD = 109%, LPS: 140%, and HMGB1: 175%) (Figure 8A, right). In MIAPaCa-2 cells, Bcl-xL expression was more intensified compared to BxPC-3 cells, and this after treatment with all TLR ligands (ODN: ROD = 291%, LTA: 204%, LPS: 170%, HMGB1: 164%, ODN + HMGB1: 339%, and LTA + HMGB1: 170%) (Figure 8B, right). PaCaDD135 cells expressed increased Bcl-xL in a range between the other two analyzed cancer cell lines (ODN ROD = 141%, LTA: 142%, LPS: 124%, HMGB1: 113%, ODN + HMGB1: 133%, and LPS + HMGB1: 110%) (Figure 8C, right).

### 2.5. TLR Ligation Induces PI3K/Akt/mTOR Signaling

**Figure 9:**
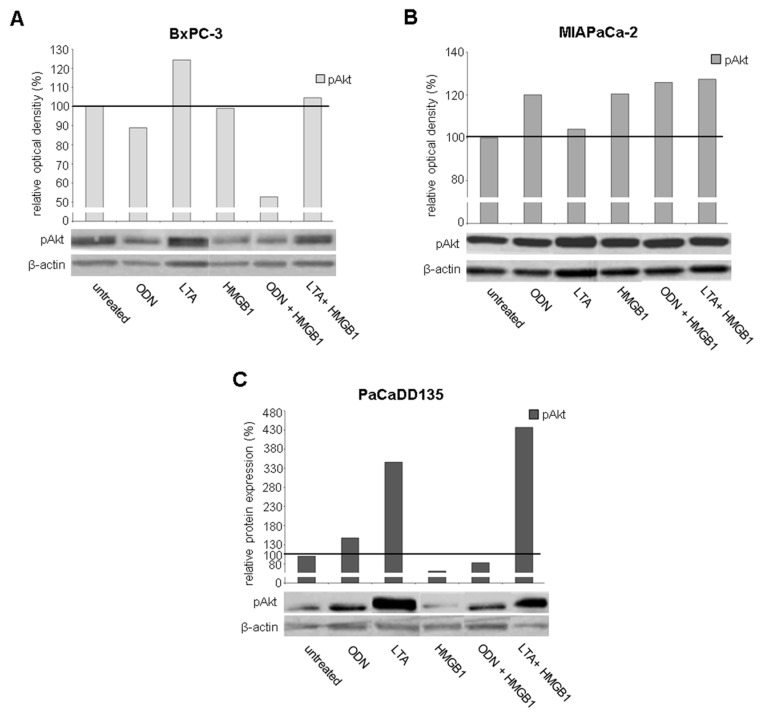
Western blot analysis of pAkt in pancreatic cancer cell lines after treatment with TLR ligands: BxPC-3 (**A**); MIAPaCa-2 (**B**); and PaCaDD135 (**C**) cells were incubated with ODN, LTA, HMGB1, ODN + HMGB1, and LTA + HMGB1 and analyzed for phosphorylated Akt after TLR activation. β-actin probe was used as control for protein loading. Relative optical density (ROD) was determined using ImageJ software: values for proteins of interest were calculated in relation to values of loading controls. Untreated cells were standardized to baseline.

Phosphorylation of the PI3K/Akt/mTOR pathway component Akt was analyzed after TLR activation by Western blot. Treatment of BxPC-3 cancer cells with LTA alone or in combination with HMGB1 resulted in an increased phosphorylation of Akt (LTA: ROD = 124%; LTA + HMGB1: ROD = 105%) (Figure 9A). MIAPaCa-2 cells demonstrated likewise elevated pAkt levels after activation with the TLR ligands either alone or when given together (ODN: ROD = 120%; LTA: ROD = 104%; HMGB1: ROD = 120%; ODN + HMGB1: ROD = 126% and LTA + HMGB1: ROD = 127%) (Figure 9B). In PaCaDD135 TLR activation resulted in much higher pAkt levels (ODN: ROD = 148%; LTA: ROD = 346%; and LTA + HMGB1: ROD = 437%) (Figure 9C).

### 2.6. TLR Activation Results in Increased Tumor Cell Proliferation

**Figure 10:**
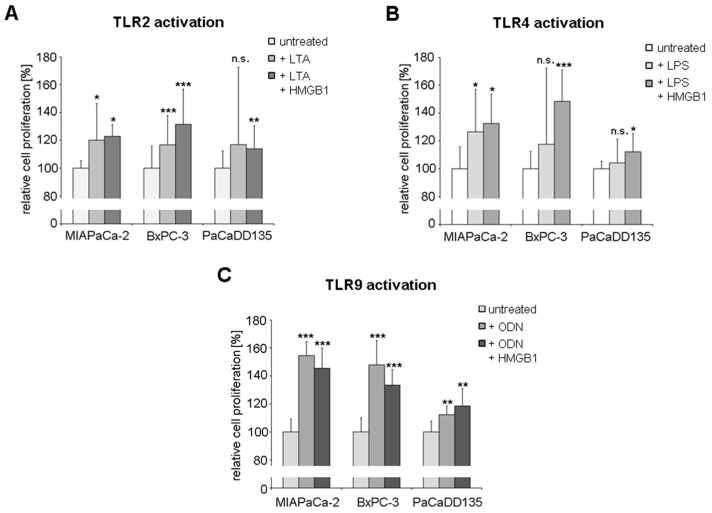
ATP proliferation assay of pancreatic cancer cell lines treated with TLR ligands. BxPC-3, MIAPaCa-2, and PaCaDD135 cells were incubated with: (**A**) LTA and LTA + HMGB1 (TLR2 activation); (**B**) LPS and LPS + HMGB1 (TLR4 activation); and (**C**) ODN and ODN + HMGB1 (TLR9 activation). Untreated cells were standardized to baseline. * *p <* 0.05, ** *p <* 0.005, *** *p <* 0.0001, n.s. not significant.

The promoting effect on proliferation through TLR2, -4, and -9 activation of MIAPaCa-2, BxPC-3, and PaCaDD135 cells was analyzed by the ATP proliferation assay. Activation of TLR2 with LTA and LTA + HMGB1 resulted in significantly increased tumor cell proliferation in MIAPaCa-2 cells (120% and 123%, *p* < 0.05), BxPC-3 cells (117% and 132%, *p* < 0.0001) and PaCaDD135 cells (117% and 114%, n.s. and *p* < 0.005) compared to untreated cells (Figure 10A).

Treatment with the TLR4 ligands LPS and LPS + HMGB1 also caused significantly increased cancer cell proliferation compared to untreated cells (MIAPaCa-2: 127%, *p* < 0.05 and 132%, *p* < 0.05; BxPC-3: 118% and 148%, *p* < 0.0001; PaCaDD135: 104% and 112%, *p* < 0.05) (Figure 10B). Interestingly, activation of TLR9 with ODN and ODN + HMGB1 showed highly significant proliferation promoting effects in MIAPaCa-2 (155% and 145%, *p* < 0.0001), BxPC-3 (147% and 133%, *p* < 0.0001), and PaCaDD135 cancer cells (112% and 118%, *p* < 0.005) (Figure 10C).

## 3. Discussion

Recently, we reported about the role of the two Toll like receptors TLR7 and -8 in solid tumor growth. Expression of the two intracellularly expressed receptors is up-regulated in primary tumors from patients with colon as well as pancreatic cancer. Interestingly, in both human cancers TLR7 and -8 expression was related rather to cancer cells and rarely detected in tumor-infiltrating immune cells. Moreover, our results indicated that TLR7 and -8 expression is associated with tumor cell growth and tumor progression in both colon and pancreatic cancer [7,8]. Here, we analyzed whether other Toll like receptors such as TLR2, -4, and -9 are additionally expressed in pancreatic cancer and may influence tumor cell signaling and proliferation to elucidate their potential for therapeutic strategies in this devastating tumor.

We demonstrated that all three receptors TLR2, -4, and -9 are constitutively expressed in the analyzed established and primary pancreatic cancer cell lines. Moreover, TLR expression in tumor cells was also detected in ex vivo tissue samples of pancreatic cancer. Consistently, in several solid human cancers, TLR expression has been assigned not only to tumor infiltrating cells of the immune system, but rather to tumor cells themselves. Interestingly, more recent data point to tumor cell located TLR2 and -9 as effective markers to distinguish cancerous from normal epithelial cells in human pancreatic cancer [16]. Our data support these observations. While primary pancreatic cancers at all UICC stages demonstrated substantial protein and gene expression of TLR2 and -9, no protein expression and only minor gene expression was observed in normal pancreatic tissue, compared to the moderate protein expression in controls with chronic pancreatitis, supporting their role in the inflammatory response. However, to this point it needs to be further analyzed whether TLR expression can be assigned not only to tumor infiltrating immune cells but significantly to tumor cells and secondly what is the functional role for this tumor cell-mediated TLR expression.

Additionally, we extended current knowledge about the expression of TLR2, -4, and -9 in the various established to non-commercially available primary human pancreatic cancer cell lines. To dissect the effects of TLR activation, we treated two of the established pancreatic cancer cell lines (BxPC-3 and MIAPaCa-2) and one primary pancreatic cancer cell line (PaCaDD135) with TLR specific ligands (LTA for TLR2, LPS for TLR4, ODN for TLR9) and a non-specific ligand (HMGB1). HMGB1 is a damage-associated inflammatory factor that plays an important role in the pathogenesis of numerous chronic inflammatory diseases [26]. It arises from inflammation and subsequent cellular injury as so called damage-associated molecular pattern (DAMP). This implies, that even in the absence of pathogens, disrupted or injured cells can create TLR ligands such as HMGB1 or nucleic acids that may activate TLR signaling when released outside the cell following tissue injury [6].

Interestingly, stimulation of TLR9 and in part TLR2 resulted in upregulated VEGF gene expression (BxPC-3) and particularly of PDGF in all three analyzed human pancreatic cancer cell lines. Moreover, significantly increased protein expression of both growth factors VEGF and PDGF was observed in all investigated cancer cells. VEGF works as a key regulator of angiogenesis and lymphangiogenesis and additionally mediates tumor cell proliferation, migration, differentiation, and cancer cell survival [27,28,29,30]. Next to VEGF, PDGF has more recently been found to be involved in migration and angiogenesis [31]. Additionally, it supports tumor cell proliferation and metabolic alterations as demonstrated in previous studies [32]. Activation of receptor tyrosine kinases, such as the PDGF receptor (PDGFR) and VEGF receptor (VEGFR), has also been implicated in tumor progression and metastasis in human pancreatic cancer [33]. VEGFR expression has been observed in about 50% of human pancreatic cancer cells [34]. Concerning PDGF receptors, high PDGFRb expression was found to be correlated with poor disease-free survival in pancreatic cancer and additionally colon and ovarian cancer patients [35]. Overall, growth factors and their receptors have been shown to result in progression and metastasis formation of pancreatic cancer and engagement of factors such as VEGF and PDGF and their signaling cascades appear to be crucial for the progression of this devastating disease [36].

After ligation with their specific receptors, both growth factors VEGF and PDGF induce subsequent receptor phosphorylation and activation of downstream signaling cascades including the PI3K/Akt/mTOR and MAPK pathway [28,31]. In our functional analysis, we observed increased phosphorylation of Erk after treatment with all TLR agonists for TLR2, -4, and -9 (except only one cancer cell line for TLR2 activation), strongly indicating activation of the MAPK signaling pathway. Moreover, upregulated phosphorylation of Akt after TLR activation, in particular TLR4, also points to an engagement of PI3K/Akt/mTOR signaling.

Notably, stimulation of all analyzed TLRs resulted in significantly increased tumor cell proliferation in all the herein investigated human pancreatic cancer cell lines. Particularly activation of TLR9 demonstrated pronounced proliferation promoting effects. Whether TLR-mediated VEGF and PDGF expression from tumor cells may lead to an autoregulative self-induction of VEGF and PDGF receptors on pancreatic cancer cells and thus acts as a feedback loop inducing tumor cell proliferation remains so far hypothetic. However, it is supported by our observed effects on both PI3K/mTOR and MAPK signaling, since both pathways are downstream signaling cascades of VEGFR and PDGFR.

In line with the proliferation promoting effects, we also demonstrated TLR-induced increase of anti-apoptotic Bcl-xL. In particular MIAPaCa-2 and PaCaDD135 pancreatic cancer cells showed considerably elevated TLR ligand-mediated Bcl-xL expression, indicating TLR signaling to induce cellular survival pathways in pancreatic cancer [37]. As described by others, a direct link between Erk activation and induction of Bcl-xL has been proposed [38]. These findings support the hypothesis that autoregulative expression of VEGF and PDGF receptors on pancreatic cancer cells may create a feedback loop to induce tumor cell proliferation and survival.

To date, a considerable number of studies suggest that TLR activation may reduce tumor growth and therefore has beneficial effects. One of the main mechanisms that explain the antitumor activity of TLR signaling is the induction of a tumor-specific immune response. TLR activation provokes the infiltration of natural killer (NK) cells, cytotoxic T cells and type I T helper cells into the tumor, where they induce tumor cell lysis via secretion of e.g., perforin and IFN-γ (interferon gamma) which results in the release of type I IFNs (IFN-α, β) [39,40,41]. Consequently, several ТLR agonists are currently in use or analyzed in clinical trials for their potential in tumor treatment. In chronic lymphatic leukemia and basal cell carcinoma, TLR7 and -8 agonists of both natural (e.g., single stranded RNA, ssRNA) and synthetic (e.g., Imiquimod) origin have demonstrated promising antitumor activity. Additionally, the TLR9 ligand CpG has been shown to suppress cancer growth in lymphomas and tumors of the brain, kidney and skin. Induction of TLR3 by its ligand poly(IC) has also been described to reduce viability in tumor cells of renal cell carcinoma and metastatic colorectal cancer [16,39,42,43,44].

Despite the numerous reports that demonstrate favorable effects of TLR stimulation, the association of about 15% of human tumors with inflammatory processes and chronic inflammation remains puzzling [17]. e.g., the association of stomach cancer with chronic inflammation induced by Helicobacter pylori is well described. Additionally, chronic inflammation of the digestive tract such as inflammatory bowel disease or chronic pancreatitis can be linked to an increased risk for colorectal or pancreatic cancer [2,39,45]. TLR signaling is known to play a major role in processes of inflammatory response and a large number of studies points to the implication of ТLR activation in tumor formation and development, angiogenesis, and tumor growth as well as chemoresistance and immune evasion by the induction of regulatory T cells (Treg) [39,46]. For example, TLR ligands demonstrated anti-apoptotic and metastatic effects in human lung cancer cells and induced proliferation in human myeloma cells (TLR3). Adhesion and metastasis in human colon cancer has been linked to TLR4 activation as well as migration in glioblastoma. TLR2 signaling has been demonstrated to induce migration in human breast cancer cells) and ligation of TLR7 and -8 has been associated with proliferation and chemoresistance in pancreatic cancer (TLR7 and -8) [8,18,19,20,21]. In the here presented study, we clearly demonstrated for the first time that ligation of TLR2, -4, and -9 results in increased tumor cell proliferation in pancreatic cancer.

Based on these observations, we conclude that inflammation-mediated cancer cell proliferation, tumor progression, and metastatic potential are closely linked to specific TLR expression and signaling in pancreatic cancer. Therefore, targeting of TLR signaling represents a key mechanism to reduce tumor growth, resistance to apoptosis and growth factor induced tumor cell proliferation. As TLR activation can be advantageous for the proliferation, invasiveness, and/or survival of tumor cells, the effects of TLR agonists on tumor cells besides tumor infiltrating immune cells depend on the tumor type, and need to be carefully taken in account in preclinical studies. Based on the current data, particularly in inflammation-mediated tumor growth and progression as is the case for pancreatic cancer, patients may finally benefit from TLR blockade rather than activation.

## 4. Materials and Methods

### 4.1. Patients and Human Tissues

In a retrospective analysis, 20 patients with histologically confirmed pancreatic cancer (adenocarcinoma) of the exocrine pancreas and available representative tumor tissues as well as completed 5-year follow up (January 2007 until December 2009) and 12 patients with chronic pancreatitis were evaluated. Patients were followed up in our Comprehensive Cancer Center (completeness index 0.96). The study was conducted in accordance with the Declaration of Helsinki. Ethical approval was obtained from the Human Research Ethics Committee of the University of Wuerzburg. All patients providing pancreatic cancer samples signed a consent form to allow for this research. The histological stage of the pancreatic cancer specimen was determined according to the UICC (Union Internationale Contre le Cancer) staging system. After acquisition, Tumor localization, stage, and differentiation grade was evaluated in our Department of Pathology. Tumor samples were snap-frozen in liquid nitrogen instantly upon surgical removal. For cryostat-sections, tissue samples were embedded in Tissue Tek (Sakura, Torrence, CA, USA) using suitable cryo molds. Samples were transferred to −80 °C until analyzed.

In our studies, we compared tissue samples of UICC stage I/II (UICC I *n* = 2, UICC II *n* = 8) and UICC stage III/IV (UICC III *n* = 7, UICC IV *n* = 3), specimens from individuals operated on histologically confirmed chronic pancreatitis (*n* = 8) and normal tissue of healthy controls (*n* = 8).

### 4.2. Cell Culture

The five human pancreatic cancer cell lines Panc1, MIA PaCa-2, BxPC-3, AsPC-1 and SW1990 were purchased from American Type Culture Collection (ATCC, Manassas, VA, USA). Panc1 cells were cultured in Dulbecco’s modified Eagle’s medium (DMEM, ATCC) supplemented with 10% (*v*/*v*) fetal bovine serum (FBS) and 1% (*v*/*v*) penicillin/streptomycin (pen/strep). MIAPaCa-2 cells were kept in DMEM (ATCC) supplemented with 10% (*v*/*v*) FBS, 2.5% (*v*/*v*) horse serum, and 1% (*v*/*v*) pen/strep. BxPC-3, AsPC-1 and SW1990 were maintained in RPMI 1640 medium (ATCC) containing 10% (*v*/*v*) FBS and 1% (*v*/*v*) pen/strep. The additional three primary pancreatic cancer cell lines PaCaDD135, PaCaDD159 and PaCaDD185 were kindly provided by Dr. Felix Rueckert (Surgical Clinic Mannheim, University of Heidelberg, Mannheim, Germany). Culture medium for primary pancreatic cancer cells lines was assembled by mixing two parts of DMEM medium supplemented with 20% (*v*/*v*) FBS with one part of Keratinocyte-SFM. All cell lines were incubated at 37 °C in 5% CO_2_.

### 4.3. Immunofluorescence Staining

The TLR2 antibody was purchased from Novus Biologicals (Littelton, CO, USA), TLR4 and TLR9 antibodies were provided by abcam (Cambridge, UK), and the antibody for EpCAM was obtained from Biorbyt (Riverside, UK). Secondary antibodies were indocarbocyanin (Cy3)-conjugated anti-rabbit and AlexaFluor488-conjugated anti-mouse IgG (both Jackson ImmunoResearch, West Grove, PA, USA). The staining was performed on serial cryostat sections of the snap-frozen specimens of pancreatic cancers and cytospin preparations of human pancreatic cancer cell lines. For immune staining procedures samples were fixed in acetone and incubated with the primary antibody in Tris-buffered saline (TBS) plus 0.5% (*m*/*v*) bovine serum albumin (BSA) overnight at 4 °C in a humidified chamber. Treatment with secondary fluorochrome conjugated antibody was performed for 30 min at room temperature in a humidified chamber. In case of double staining, slides were incubated with the second primary antibody diluted in TBS plus 0.5% (*m*/*v*) BSA overnight at 4 °C in a humidified chamber followed by incubation with secondary fluorochrome conjugated antibody for 30 min at room temperature in a humidified chamber. Subsequently, slides were mounted with 4′,6-diamidin-2-phenylindol (DAPI) Fluoromount-G (Southern Biotech, Birmingham, AL, USA) and analyzed using an Olympus BX51 microscope and the CellSens Dimension software (Olympus, Hamburg, Germany).

### 4.4. In Vitro Activation of TLR2, -4, and -9 

To investigate the effects of TLR activation BxPC-3, MIAPaCa-2, and PaCaDD135 cells were treated with the TLR ligands oligodeoxyribonucleotide2006 (ODN; TLR9 activation), lipoteichonic acid of Staphylococcus aureus (LTA; TLR2), lipopolysaccharides (LPS; TLR4) and high-mobility group box 1 (HMGB1; non-specific). ODN2006, LTA-SA and LPS were obtained from Invivogen (San Diego, CA, USA), HMGB1 was provided by abcam. Cells were cultured for up to 96 h with daily stimulation using 1 μg/mL LTA, 1 µg/mL LPS, 2.5 µg/mL ODN, and/or 1 µg/mL HMGB1. Then cells were detached using accutase solution (Sigma-Aldrich, St. Louis, MO, USA) and extraction of total RNA or proteins was performed.

### 4.5. Quantitative Real Time RT-qPCR

Gene expression for TLR2, -4, and -9 as well as VEGF and PDGF was determined using quantitative real-time PCR (RT-qPCR). For analysis of pancreatic tissue samples human pancreatic matched cDNA was purchased from Pharmingen (Heidelberg, Germany) and used as a control. Total cellular RNA was extracted using RNeasy Minikit (Qiagen, Hilden, Germany) on the QIAcube platform (Qiagen) according to the manufacturer’s instructions. Reverse transcription was performed using ImProm-II reverse transcriptase system (Promega, Mannheim, Germany) and carried out on a Eppendorf Mastercycler (Eppendorf, Hamburg, Germany). qPCR was performed using Taqman Gene Expression Master Mix (Life Technologies, Carlsbad, CA, USA) and Taqman Gene Expression Assays (Life Technologies) in concordance to the manufacturer’s instructions and carried out in duplicates on a Biorad CFX96 Touch Real-Time PCR Detection System. Reproducibility was assured by three independent measurements. Housekeeping genes β-actin, glycerinaldehyd-3-phosphat-dehydrogenase (GAPDH), and β2-microglobulin (B2M) were used for calculation of the relative quantification value, fold difference, which is expressed as 2^−ΔΔ*C*q^.

### 4.6. Western Blot

Protein extraction from tissue samples and pancreatic cancer cell lines was performed using RIPA buffer containing dithiothreitol (DTT) and protease/phosphatase inhibitor cocktails (Merck Millipore, Billerica, MA, USA). Tumor and normal tissues were cut in small pieces and homogenized for 10 min in prepared RIPA buffer using TissueLyser (Qiagen) before centrifugation (full speed, 4 °C, 20 min). The supernatant was collected and stored at −80 °C.

For preparation of cell lysates adherent cells were detached using accutase solution, washed with Dulbecco’s phosphate buffered saline (DPBS, Thermo Fisher Scientific, Waltham, MA, USA) and pelleted at 300× *g* for 10 min. After resuspension in prepared RIPA buffer, cells were incubated on a rotator at 4 °C for 10 min, then centrifuged (full speed, 4 °C, 20 min) and supernatant was collected and transferred to −80 °C. To determin the protein concentration of the prepared lysates Bradford assay was performed using Roti-Quant solution (Carl Roth, Karlsruhe, Germany). For gel electrophoresis, NuPAGE SDS Buffer and NuPAGE Novex Mini Gels (Thermo Fisher Scientific) were used and handled according to the manufacturer’s instructions. Western blotting on nitrocellulose was performed on the iBlot dry Blotting System using iBlot Gel Transfer Stacks (Thermo Fisher Scientific) according to the manufacturer’s instructions.

Blots were probed with antibodies against TLR2, TLR4, TLR9, β-actin, pAkt, pErk, cofilin and Bcl-xL. Antibodies against TLR2, TLR4, and Bcl-xL were obtained from abcam. β-actin, cofilin, pAkt, and pErk antibodies were purchased from Cell Signaling Technology (Danvers, MA, USA). TLR9, anti-mouse IgG and anti-rabbit IgG horseradish peroxidase (HRP)-conjugated secondary antibodies were obtained from Santa Cruz Biotechnology (Dallas, TX, USA).

### 4.7. Flow Cytometry

Pancreatic cancer cell lines BxPC-3, MIAPaCa-2, and PaCaDD135 were analyzed for the expression of TLR2, -4, and -9 using a flow cytometer (Beckman Coulter, Krefeld, Germany) with a software package Coulter Epics XL-MCL, System II (Beckman Coulter). Phycoerythrin (PE)-conjugated TLR2 and TLR4 antibodies as well as isotype control antibodies were purchased from Miltenyi (Bergisch Gladbach, Germany), PE-conjugated antibody against TLR9 was provided by eBioscience (San Diego, CA, USA). For intracellular staining we used Intraprep-Kit (Beckman Coulter). Staining was performed according to the manufacturers’ instructions.

### 4.8. Luminex Analysis

Cells were seeded (1.5 × 10^5^) in 6-well plates and treated with LTA, ODN, and/or HMGB1 after pre-incubation for 3 days. Supernatants were collected 24 h and 48 h after TLR activation. To determine the protein expression of VEGF and PDGF, Luminex analysis was accomplished using Milliplex Map Kit (Merck Millipore, Darmstadt, Germany) according to the manufacturer’s instructions.

### 4.9. ATP Cell Proliferation Assay

To investigate the effect of stimulation with TLR ligands on tumor cell proliferation of BxPC-3, MIAPaCa-2, and PaCaDD135 cells, 6000 cells/well were seeded in 96-well plates and pre-incubated for 3 days following daily stimulation with LTA-SA, LPS-B5, ODN2006, and/or HMGB1 for additional 4 days. Then, 24, 48, 72, and 96 h after activation, ATP proliferation assay (ATP Determination Kit, Thermo Fisher Scientifc) was performed according to the manufacturer’s instructions.
